# Insights into preeclampsia: a bioinformatics approach to deciphering genetic and immune contributions

**DOI:** 10.3389/fgene.2024.1372164

**Published:** 2024-08-06

**Authors:** Rongrong Zhong, Yifen Guo, Jianxing Huang, Yingao Yang, Shuyue Ren, Yan Gu, Ping Lei, Zhixian Gao

**Affiliations:** ^1^ Deparment of Geriatrics, Tianjin Medical University General Hospital, Tianjin, China; ^2^ Department of Family Planning, The Second Hospital of Tianjin Medical University, Tianjin, China; ^3^ Tianjin Key Laboratory of Risk Assessment and Control Technology for Environment and Food Safety, Tianjin Institute of Environmental and Operational Medicine, Tianjin, China; ^4^ Medical Imaging and Nuclear Medicine, Jinzhou Medical University, Jinzhou, Liaoning, China

**Keywords:** preeclampsia, Mendelian randomization, single-cell RNA sequencing, expression quantitative trait loci, GWAS

## Abstract

**Background:**

Preeclampsia (PE) is a global pregnancy concern, characterized by hypertension with an unclear etiology. This study employs Mendelian randomization (MR) and single-cell RNA sequencing (scRNA-seq) to clarify its genetic and molecular roots, offering insights into diagnosis and treatment avenues.

**Methods:**

We integrated PE-specific genome-wide association study (GWAS) data, expression and protein quantitative trait loci (eQTL and pQTL) data, and single-cell data from peripheral blood mononuclear cells (PBMCs). We identified highly variable genes using single-cell information and employed MR to determine potential causality. We also combined pQTL and GWAS data, discerned genes positively associated with PE through scRNA-seq, and leveraged the Enrichr platform to unearth drug-gene interactions.

**Results:**

Our scRNA-seq pinpointed notable cell type distribution variances, especially in T helper cells (Th cells), between PE and control groups. We unveiled 591 highly variable genes and 6 directly PE-associated genes. Although MR revealed correlations with PE risk, pQTL analysis was inconclusive due to data constraints. Using DSigDB, 93 potential therapeutic agents, like Retinoic acid targeting core genes (IFITM3, NINJ1, COTL1, CD69, and YWHAZ), emerged as prospective multi-target treatments.

**Conclusion:**

Utilizing MR and scRNA-seq, this study underscores significant cellular disparities, particularly in Th cells, and identifies crucial genes related to PE. Despite some limitations, these genes have been revealed in PE’s underlying mechanism. Potential therapeutic agents, such as Retinoic acid, suggest promising treatment pathways.

## 1 Introduction

Preeclampsia (PE) is a complex pregnancy-specific hypertensive disorder that manifests after 20 weeks of gestation (Burton et al., 2019; Aneman et al., 2020). It accounts for 4.6% of pregnancies worldwide (Abalos et al., 2013), with its incidence sharply rising over the past 30 years (Opichka et al., 2021). PE is characterized by hypertension, proteinuria, and multi-system involvement and can develop into complications such as eclampsia or HELLP syndrome. PE not only impairs fetal brain development, increasing the risk of intellectual disabilities, autism and schizophrenia (Dachew et al., 2018; Ursini et al., 2018), but also contributes to premature birth and fetal malformations (Weiler et al., 2011). Thus, continuous research is essential to improve outcomes for affected mothers and their children (ACOG, 2019).

The etiology of PE is complex and remains not fully understood. Previous studies have established associations between PE and factors such as endothelial dysfunction, intravascular inflammation, syncytiotrophoblast stress, placental aging, and the breakdown of maternal-fetal immune tolerance (Johnsen et al., 2018). The pathogenic mechanisms include failure of spiral artery remodeling (Staff et al., 2022), imbalance of vascular endothelial growth factor and sFlt1 (Vieillefosse et al., 2016), placental oxidative stress (Guerby et al., 2021), immune dysregulation (Wang et al., 2013), and progressive deterioration that can only be cured by delivery (Gestational Hypertension and Preeclampsia, 2020). A deeper understanding of how genetic factors contribute to PE is essential for enhancing diagnosis and treatment levels.

Mendelian randomization (MR) is an innovative statistical method that utilizes genetic variations as instrumental variables to assess causal relationships between exposures and outcomes (Grover et al., 2017). Mendelian principles of randomly assigning gene variants during conception enable MR to effectively reduce confounding bias and reverse causality, enhancing its utility for dissecting complex genetic and environmental interactions in diseases (Verduijn et al., 2010). Its inherent ability to reduce bias underscores its reliability and critical role in the field of genetic epidemiology (Bowden and Holmes, 2019). In recent years, the application of MR has expanded into areas such as the gut microbiome, the relationship between obesity and female reproductive conditions, and the effects of antihypertensive drugs on pregnancy-related diseases. For instance, a two-sample MR study by Pengsheng Li and colleagues identified a causal relationship between Bifidobacteria and the onset of preeclampsia-eclampsia (Pengsheng et al., 2022); genetic analyses by Samvida S Venkatesh et al. revealed heterogeneous associations between overall and central obesity and risks of reproductive disorders (Samvida S et al., 2022); research in the Mendelian randomization paradigm by Maddalena Ardissino et al. suggested that the use of BBs can reduce birth weight, while CCBs can decrease the risk of preeclampsia and eclampsia without affecting the risk of gestational diabetes or birth weight (Maddalena et al., 2022). These studies further demonstrate the efficacy and broad applicability of the MR approach across multiple fields.

Furthermore, single-cell RNA sequencing (scRNA-seq) permits a detailed analysis of gene expression in individual cells, revealing molecular heterogeneity (Tanay and Regev, 2017). Techniques integrating scRNA-seq with Mendelian analysis have revealed complex patterns of gene expression and genetics, allowing researchers to identify gene loci that influence expression, understand patterns of transgenerational inheritance, and elucidate the molecular regulatory mechanisms underlying PE (Visscher et al., 2017). In this study, we utilized MR methods, tissue-specific quantitative trait loci (QTL) data, and scRNA-seq to deeply explore the etiology and pathogenic mechanisms of PE. Our findings highlighted the importance of core genes IFITM3, NINJ1, COTL1, CD69, and YWHAZ in PE’s etiology and proposed retinoic acid as a viable therapeutic candidate for PE, effectively linking molecular discoveries to potential clinical treatments.

## 2 Methods

### 2.1 Study design

**FIGURE 1:**
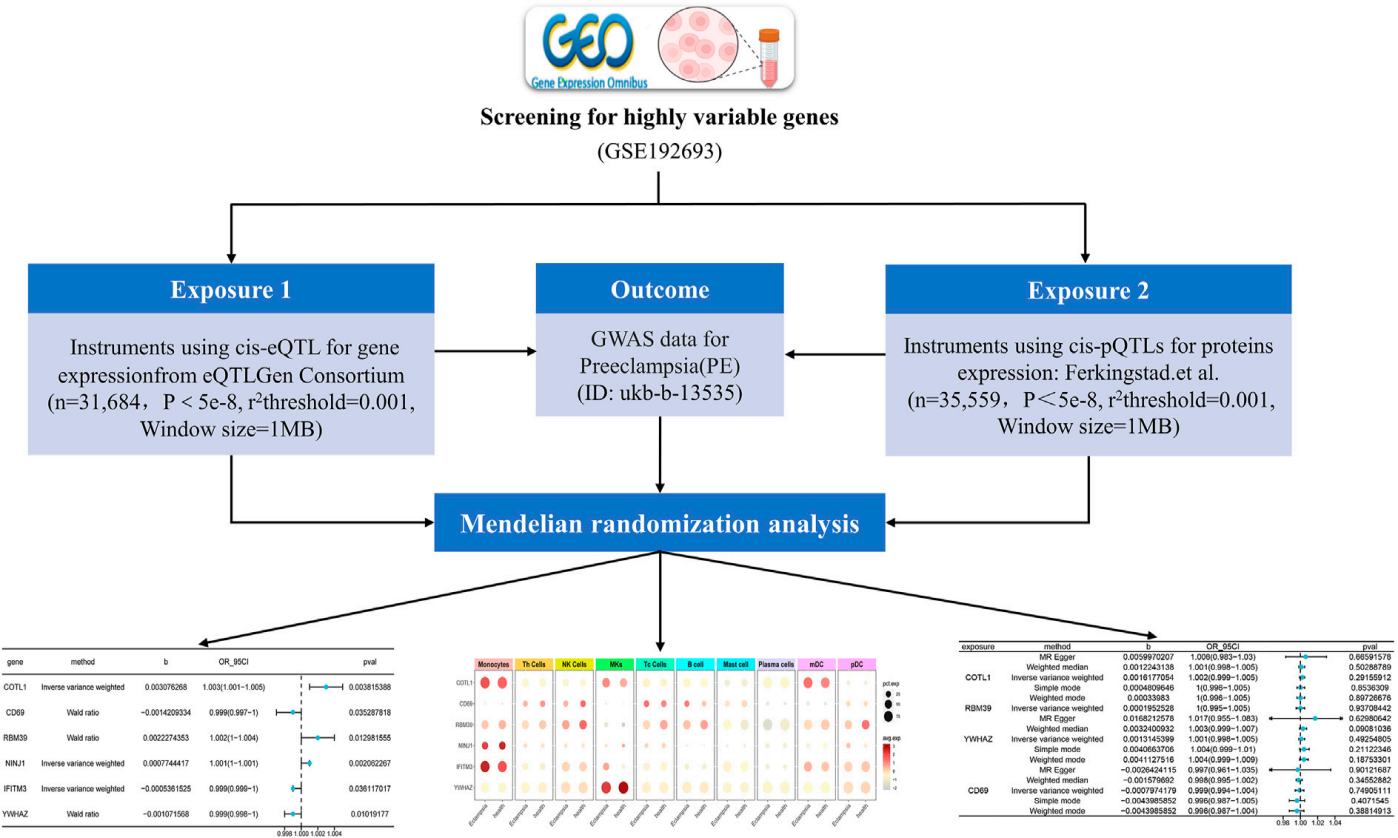
Overview of the MR Analysis Framework in This Study: The flow chart illustrates the step-by-step process through which the MR analysis was systematically conducted in this research (eQTL: expression quantitative trait loci; GWAS: genome-wide association study; pQTL: protein quantitative trait loci).

The effectiveness of MR analysis is contingent upon three critical assumptions. Firstly, the selected genetic variants must be significantly associated with the exposure; secondly, these variants, as instrumental variables, should not be associated with confounding factors, ensuring their independence; finally, the genetic variants should influence the outcome exclusively through the exposure, without any horizontal pleiotropic effects. These assumptions ensure the integrity and dependability of MR for investigating causal inferences (Zheng et al., 2017). In this study, we began by identifying genes with high variability via scRNA-seq. We then utilized MR, using the expression quantitative trait loci (eQTL) data of these genes as exposures and PE as the outcome to identify relevant genes. Following this, we obtained protein quantitative trait loci (pQTL) data for these identified genes and performed additional MR analysis using the genome-wide association study (GWAS) data related to PE. Finally, by integrating single-cell sequencing data, we determined the cellular distribution of the significant genes in relation to PE. The experimental process was depicted in Figure 1.

Rationale for Selection of Genes and Cell Types: Genes with high variability in scRNA-seq data were selected to capture those most likely to exhibit significant associations with PE. This approach focuses on identifying key players in the disease mechanism. Similarly, specific cell types, particularly immune cells, were targeted due to their known involvement in PE pathogenesis.

### 2.2 Source of PE GWAS data

We sourced the GWAS dataset pertinent to PE from the IEU Open GWAS project (https://gwas.mrcieu.ac.uk/), designated with the ID number ukb-b-13535. Assembled in 2018, this dataset predominantly comprised genetic information from the European demographic. It encompassed data from 1,864 individuals diagnosed with PE and 461,069 controls. An extensive analysis within this dataset scrutinized 9,851,867 single nucleotide polymorphisms (SNPs), facilitating a comprehensive investigation into the genetic determinants of PE.

### 2.3 Source of quantitative trait locus data

For the investigation of eQTLs in blood tissues, summary statistics were acquired from the eQTLGen consortium (https://www.eqtlgen.org/cis-eqtls.html). Our analysis concentrated on cis-eQTLs that exhibit a strong association with gene expression, confined to a 1-Mb window flanking the coding sequences. The dataset comprised data for 10,317 SNPs and gene expression levels of 19,942 genes across the entire set of 31,684 blood samples that had been sequenced (Võsa et al., 2021). Notably, eQTLGen did not encompass variations associated with gene expression levels on the X and Y chromosomes and mitochondrial DNA (mtDNA).

To delve into the complex relationships between plasma protein levels and specific genetic loci, we employed a linear mixed model for pQTL analysis. Linear mixed models not only revealed associations between pQTL and protein levels but also took into consideration potential confounding factors and correlations between samples. Our data originated from the GWAS conducted by Ferkingstad, E. et al., which studied plasma proteins in 35,559 Icelanders and analyzed 4,907 aptamers (Mark et al., 2009). The dataset was accessible at https://www.decode.com/summarydata/.

### 2.4 Source of PE peripheral blood mononuclear cell single-cell sequencing data

In the methodology of this study, we referred to the dataset GSE192693 from the GEO database. This dataset, compiled by HU J et al., included scRNA-seq analysis of 80,429 cells from 6 patients with PE and 4 healthy controls. It primarily encompassed various immune cell subsets within peripheral blood mononuclear cells (PBMCs), such as T cells, B cells, NK cells, and Myeloid cells. Through our analysis, we aimed to explore the immunological response patterns associated with PE throughout pregnancy and compare them with those in a healthy control group. Our objective was to delineate the immunological alterations and the underlying mechanisms in PE, thereby laying the groundwork for further causal inference and the development of potential therapeutic strategies.

### 2.5 Single-cell analysis of PE peripheral mononuclear cells

Quality Control: We utilized R (v.4.1.2) and the Seurat package (v.4.3.0) for scRNA-seq data analysis. Initial quality control filtered out cells with gene detection counts outside the range of 200–10,000 and cells with mitochondrial genes exceeding 20% or hemoglobin genes over 3%. These metrics were displayed through violin plots. Normalization procedures included “LogNormalize” and “SCTransform” methods, targeting 10,000 as a scaling factor. Subsequently, the top 2,000 highly variable genes were identified for principal component analysis (PCA) reduction. Clustering and dimensionality reduction were performed using the UMAP technique and the “FindClusters” function, respectively. For marker gene expression, potential markers for each cell cluster were identified using Seurat’s “FindAllMarkers” function. These genes, differentially expressed across clusters, were manually reviewed for biological relevance and consistency with known cell-type-specific markers, by referencing existing literature and databases. This manual verification focused on genes with clear expression patterns specific to cell clusters and known roles in cell identity and function. This step validated the cell cluster labels derived from statistical analysis, ensuring they accurately reflected the distinct cell populations in PBMCs from PE patients and controls.

Differential Gene Selection: In the process of analyzing differentially expressed genes (DEGs), we employed the “FindMarkers” function in Seurat for cell type-specific differential expression analysis. This analysis utilized a two-sided Wilcoxon rank-sum test to identify genes showing significant expression differences when comparing 6 patients with PE to 4 healthy controls, based on an adjusted *p*-value below 0.05 and an absolute log2FC greater than 0.3.

### 2.6 Two-sample MR analysis

In our research methodology, we employed a MR approach to investigate a possible causal relationship between the highly variable genes screened at the single-cell level and the ukb-b-13535 phenotype. Specifically, the MR method utilized genetic variants strongly associated with exposure factors as instrumental variables (IV), thereby evaluating the causal effect between exposure factors and the study outcome. Utilizing MR enabled us to avoid interference from common problems in traditional observational studies, such as confounders, reverse causation, and measurement errors.

To conduct the MR analysis, we first ensured consistency in exposure and outcome data regarding reference alleles, effect alleles, and direction, and made necessary adjustments. We then completed matched reference and effect alleles during the SNPs selection phase. By employing the primary MR analysis function of the “GagnonMR” package, we conducted a detailed MR analysis for each SNP-phenotype pair to discern the causal impact of the DEGs’ eQTLs on the ukb-b-13535 phenotype. Finally, we visualized the DEGs with significant causal effects (*p* < 0.05) through forest plots and bar graphs.

### 2.7 Statistical analysis

All analyses were performed using R (version 4.1.2, www.r-project.org). For constructing MR estimates, we utilized the “TwoSampleMR” R package (version 0.5.7) (Hemani et al., 2018). A suite of advanced MR techniques—Inverse Variance Weighting (IVW), MR-Egger, Weighted Median, and Mode-Based Estimation—were employed to rigorously calculate effect sizes, standard errors, and P-values. We utilized Seurat’s “FindMarkers” for cell-specific DEG analysis using a two-sided Wilcoxon test. We assessed the statistical power of our MR analyses by considering a comprehensive set of parameters: sample size, a significance threshold of 0.05, the variance explained (R^2^) in the exposure by the instrumental variables, and the case-control ratio, while ensuring that the core assumptions of MR were met.

### 2.8 Drug-gene interactions reveal potential therapeutic targets

The DSigDB was a comprehensive database encompassing numerous drug-gene interactions and drug-effect signatures. We utilized the online platform Enrichr (https://amp.pharm.mssm.edu/Enrichr/) to access the DSigDB (Drug Signatures Database), facilitating the identification of drugs potentially relevant to our research targets. By comparing our experimental results with DSigDB’s drug signatures, we were able to pinpoint promising drug candidates for further study. Enrichr’s integration of various drug-gene interaction databases significantly enhanced the efficiency and accuracy of our analysis.

## 3 Results

### 3.1 Identification of 591 DEGs between healthy controls and PE samples

**FIGURE 2:**
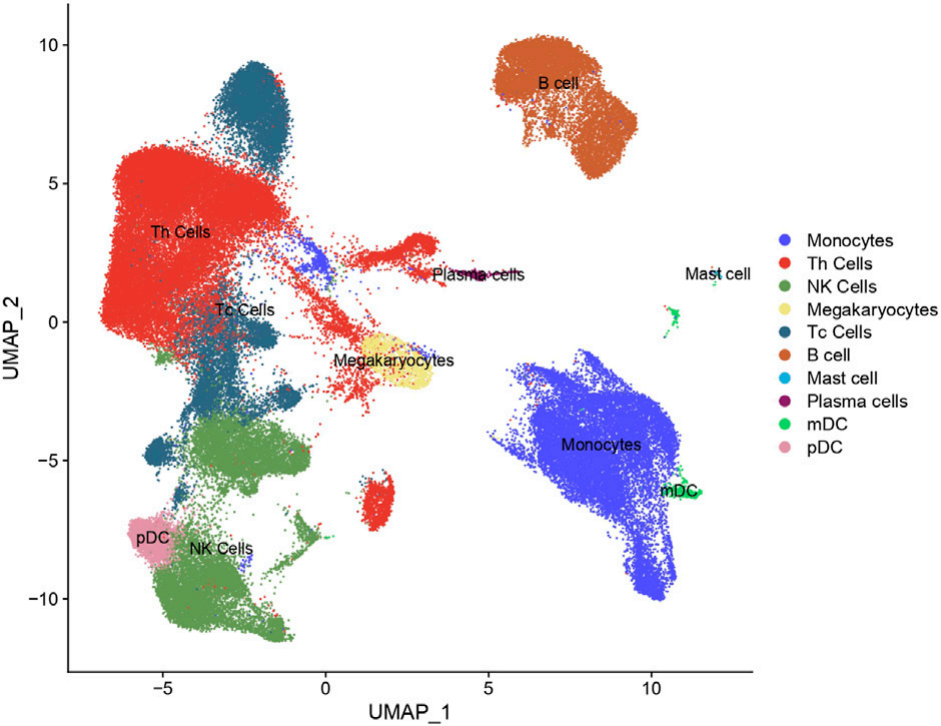
Cluster annotation and cell type identification by means of UMAP. All cells were classified into ten types and displayed across two UMAPs.

**FIGURE 3:**
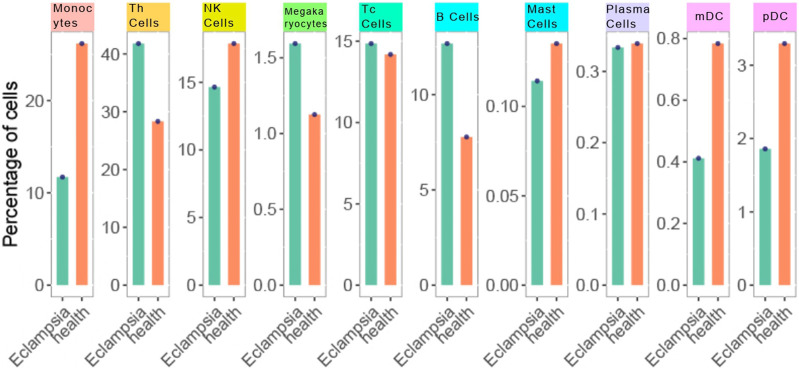
Cell type distribution in PE vs Healthy control samples. Each bar represents the percentage of cells within a sample. The orange bars denote the PE samples, while the green bars represent the healthy controls. Data points overlaying the bars indicate the actual percentage values obtained from cell counting.

In our study, scRNA-seq enabled the categorization of cells into ten distinct types through detailed clustering and analysis of marker gene expression. This approach effectively identified various immune cell populations, such as T helper cells (Th cells), Monocytes, and B cells, which were further depicted in the UMAP plot (Figure 2). By leveraging these technologies, we gained a comprehensive understanding of the immune landscape in PE patients. We then investigated the immune profiles of patients with PE in comparison to healthy subjects. Figure 3 showed that individuals diagnosed with PE had increased proportions of Monocytes, Th cells, and Plasmacytoid dendritic cells (pDCs) compared to healthy subjects, while the abundance of B cells was marginally reduced in the eclamptic cohort. Other immune cells, including Natural killer cells, Cytotoxic T cells, Megakaryocytes, Mast cells, and Plasma cells, showed no significant differences between the two groups, indicating that the immune cell perturbations in PE were cell type-specific.

**FIGURE 4:**
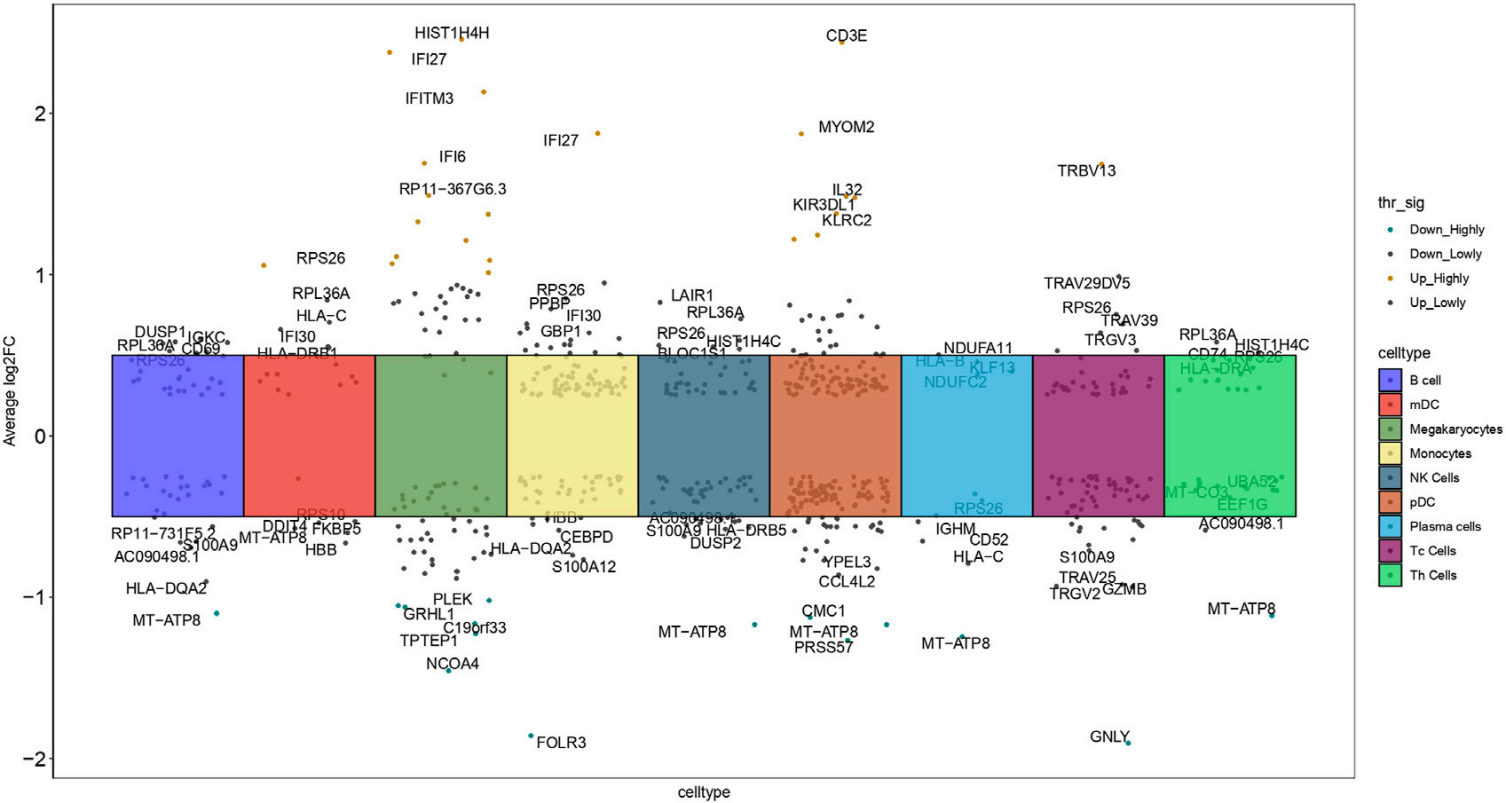
Manhattan plot depicted 591 DEGs across nine cell types, contrasting healthy controls with PE samples, and highlighted the top five upregulated and bottom five downregulated genes in each cell type (log2FC: log2 fold change).

Based on this cell type categorization, our analysis revealed 591 genes with differential expression specifically related to these cell types in PE patients compared to healthy controls, identified based on stringent criteria: an adjusted p-value of less than 0.05 and an absolute average log2 fold change (avg log2FC) greater than 0.3. These criteria ensured robust identification of genes significantly altered in PE. For each cell type, we highlighted the top five upregulated and bottom five downregulated genes with the largest log2FC, each annotated with its respective gene symbol (Figure 4). This detailed gene profiling provided a clearer picture of the molecular mechanisms underlying PE. These DEGs may provide insights into the molecular mechanisms underlying PE, offering potential targets for therapeutic intervention.

### 3.2 MR analysis of gene quantity trait loci

**FIGURE 5:**
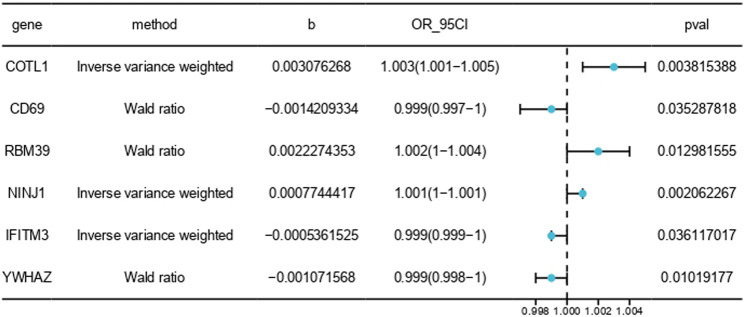
MR results between blood tissue gene expression and the risk of PE onset. The symbol “b” denotes the effect size (β) of the variant site on mRNA expression. A β > 0 indicates a positive correlation, whereas β < 0 indicates a negative correlation. The odds ratio (OR) is computed from the causal estimate of the expected value (β coefficient). The 95% confidence interval (95CI) is calculated using β and the standard error (SE).

In exploring the pathogenic genes associated with PE, we applied MR to integrate and analyze summary data from GWAS and eQTL related to PE. MR analysis was employed to infer potential causal relationships between gene expression and PE risk, minimizing biases common in observational studies. This MR analysis aimed to elucidate potential genetic contributors to the pathogenesis of PE by examining the association between gene expression and disease risk. For this analysis, we specifically focused on six genes: COTL1, CD69, RBM39, NINJ1, IFITM3, and YWHAZ, selected based on their significant differential expression in PE patients and their potential roles in immune response and placental function. We assessed their MR effect sizes (b), odds ratios (OR), 95% confidence intervals (CI), and P-values (pval) (Figure 5).

Our analysis demonstrated that higher expression levels of CD69, IFITM3, and YWHAZ were associated with a reduced risk of PE, suggesting a protective effect against the disease. Conversely, increased expression of COTL1, RBM39, and NINJ1 was correlated with a heightened risk, indicating their potential roles in exacerbating PE. These findings highlight the complex genetic architecture of PE and underscore the importance of specific genes in its pathogenesis.

### 3.3 MR analysis of pQTL

**FIGURE 6:**
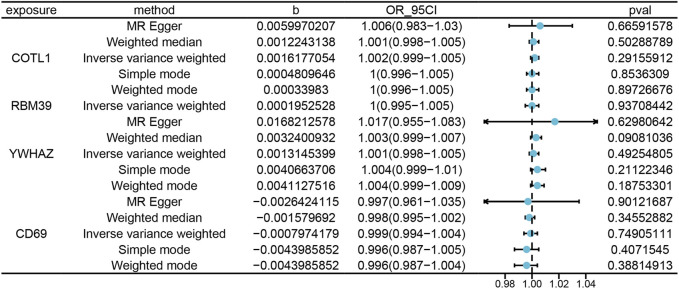
Illustrates the MRresults correlating blood tissue protein expression with the risk of PE onset. “Exposure” refers to the protein name, and “Method” indicates the specific statistical or computational method utilized. The variable “b” represents the effect size (β) of the variant site on mRNA expression, with β > 0 indicating a positive correlation and β < 0 a negative correlation. The Odds Ratio (OR) and its 95% confidence interval (OR_95CI) are derived from the causal estimate of the expected value (β coefficient), with the 95CI computed using β and the standard error (se). The p-value (Pval) is included as a measure for statistical hypothesis testing.

In our analysis, we explored protein-expression dynamics in PE by examining cis-pQTL data from key genes, including COTL1, RBM39, YWHAZ, and CD69. Protein-level analysis provides another layer of insight into the biological processes involved in PE. However, our MR analysis did not identify any causal relationships. A potential reason for this could have been the underutilization of the current pQTL dataset, with few identified genetic variations closely related to protein levels in PE (Figure 6). The lack of comprehensive pQTL data highlights a significant gap in current research, emphasizing the need for more extensive datasets to fully understand the protein-level changes in PE. This finding underscored the need for more comprehensive pQTL data to enhance our understanding of the molecular mechanisms underlying PE.

### 3.4 Single-cell sequencing analysis to determine gene distribution

**FIGURE 7:**
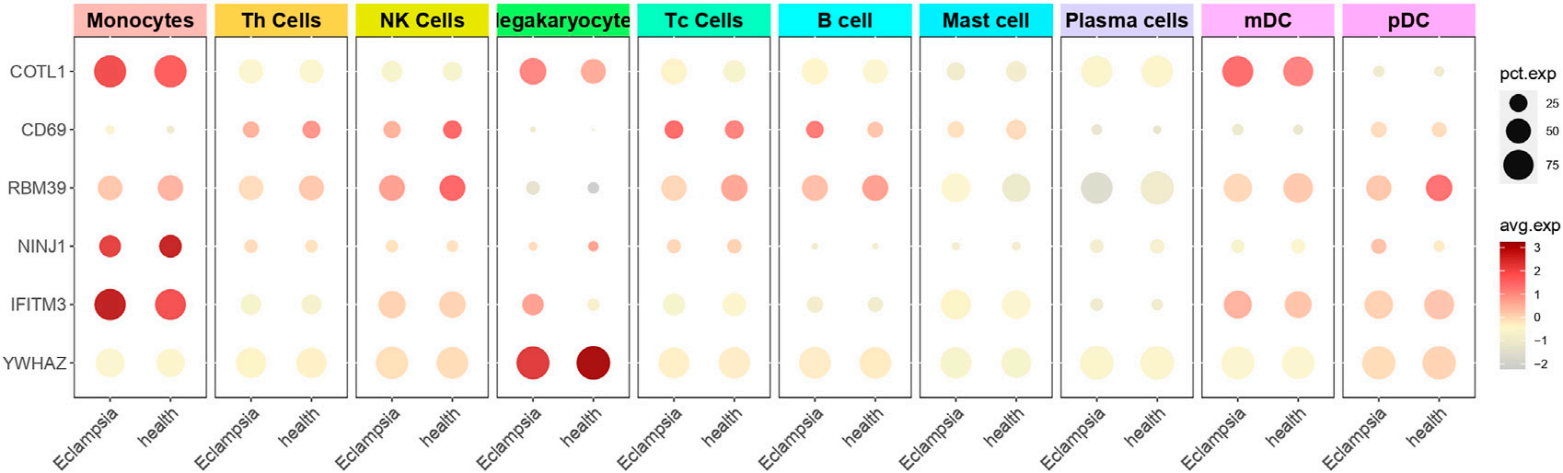
Single-cell sequencing to determine gene cell distribution. The figure presented a dot plot that depicted differential gene expression in various cell types between preeclampsia and healthy control samples. Each row represented a specific gene, and columns corresponded to cell types such as Monocytes, T Cells, and B Cells. Dot sizes indicated the percentage of cells expressing each gene, while color intensity reflected the average expression level.

To investigate the role of genes in PE within immune cells, single-cell sequencing was utilized to evaluate gene expression in PBMCs, and the findings were detailed in Figure 7. COTL1, a gene associated with PE pathogenesis, showed significant overexpression in Monocytes, Megakaryocytes, and Myeloid dendritic cells (mDCs) in the PE group, indicating its substantial role in disease processes. In contrast, RBM39, also implicated as a pathogenic gene, was predominantly expressed in a variety of immune cells including Monocytes, Th cells, NK cells, Tc cells, B cells, mDCs, and pDCs in healthy individuals, suggesting its regulatory function in immune homeostasis, which might have been disrupted in PE. Similarly, NINJ1, identified as pathogenic, was found to have had increased expression in Monocytes in the PE group, suggesting a potential monocyte-centric mechanism in the disease. The consistent expression of non-pathogenic genes CD69 and IFITM3 in both PE and healthy cohorts indicated a baseline immune activation, while YWHAZ, also non-pathogenic, was specifically identified in Megakaryocytes within the healthy group. These distinct expression patterns of pathogenic and non-pathogenic genes in various immune cells emphasize their differential roles in the immune response and the progression of PE. The distinct expression patterns of these genes in various immune cells underscored their potential roles in the dysregulated immune response observed in PE.

### 3.5 Predictive drugs for pathogenic genes

In our comprehensive analysis using DSigDB, we identified 93 drugs with therapeutic potential for PE, as listed in Supplementary Table S1. Among these, Retinoic acid was selected for focused discussion. Retinoic acid, known as a metabolite of Vitamin A, played a crucial role in immune regulation and cellular differentiation, both of which were vital in the development of PE (Pawlikowski et al., 2000; Rajakumar et al., 2020). Research revealed that the modulation of immune responses and placental development by Retinoic acid was essential in PE, with abnormalities in its signaling pathways leading to immune dysregulation and affecting key genes like IFITM3, NINJ1, COTL1, CD69, and YWHAZ. These insights suggest that Retinoic acid could be a promising therapeutic candidate for PE, warranting further investigation in clinical settings. Our analysis found that these genes were significant in the pathogenesis of PE. The ability of Retinoic acid to influence the expression of these genes aligned with our findings and highlighted its potential utility in modulating key aspects of PE pathogenesis.

## 4 Discussion

In this study, we embarked on a systematic analysis of immune cell subgroups within PBMCs by employing scRNA-seq data sequencing technology, aiming to elucidate the molecular and cellular-level characteristics of PE. This investigation identified 10 major immune cell subgroups and revealed 6 genes significantly associated with PE: COTL1, CD69, RBM39, NINJ1, IFITM3, and YWHAZ. The expression patterns of these genes within specific cell subgroups underscored their potential central roles in the pathological processes of PE. Notably, the expressions of CD69 and COTL1 were correlated with a decrease and increase in the risk of PE, respectively. Furthermore, MR analysis and differential expression detection reinforced these findings. Our results proposed retinoic acid as a viable therapeutic candidate for PE, effectively linking molecular discoveries to potential clinical treatments.

The precise etiology and pathogenesis of PE remained not fully understood, though some factors related to its development had been identified. Due to this uncertainty, its prevention and treatment strategies continued to be debated (Ives et al., 2020). In our study, we identified various cell types in PE through scRNA-seq, including Th cells, Monocytes, Megakaryocytes, etc. Particularly noteworthy was the largest proportion of Th cells in our samples. Existing studies had suggested that PE patients might alter the normal T-lymphocyte response, especially the ratio increase of Th1/Th2 lymphocytes, with Th1 cell totals similar to non-pregnant women and a cytokine profile leaning towards pro-inflammatory cytokines like IFNγ and IL-4 (Hashemi et al., 2017). Concurrently, regulatory T cell numbers had also been reduced in the placental bed and chorionic membrane of PE patients (Boij et al., 1989). Moreover, in the process of Megakaryocyte formation and platelet homeostasis in preterm infants born to PE patients, soluble FMS-like tyrosine kinase 1 (sFlt1) had been found to play a crucial role (Yang et al., 2016). The proportion of CD14^+^ Monocytes also increased in women in the prepartum or postpartum period of PE (Brien et al., 2019), further emphasizing the key role of Monocytes as the most common cell type in the circulating immune system in the disease. Our research, through analysis down to the single-cell level, had confirmed many previous perspectives regarding PE-related cellular immune responses and further revealed the functions and roles of these immune cells in PE.

By integrating the MR method with GWAS and expression eQTL analysis in PE, we confirmed six genes significantly associated with PE: COTL1, CD69, RBM39, NINJ1, IFITM3, and YWHAZ. Specifically, the expressions of CD69, IFITM3, and YWHAZ were negatively correlated with the risk of PE. The suppressive function of CD69 gene had been observed in TH17-mediated immune responses (Martín et al., 2010), possibly explaining its role in reducing the risk of PE. The IFITM3 gene was involved in cell adhesion, migration, proliferation, and immune response (Siegrist et al., 2011), consistent with previous research on the regulation of cell adhesion molecules in PE pathophysiology (Jung et al., 2022). Furthermore, the YWHAZ gene, related to cell cycle and proliferation regulation (Zhao et al., 2018), might have revealed its precise role in the disease mechanism in connection with PE’s cell cycle abnormalities and trophoblastic cell over-proliferation (Fishel Bartal and Sibai, 2022), offering intriguing perspectives for future research.

In contrast to the genes CD69, IFITM3, and YWHAZ, the expression levels of COTL1, RBM39, and NINJ1 were positively correlated with the risk of PE. The COTL1 gene encoded an essential actin-binding protein, playing a critical role in maintaining cell morphology and regulating vascular structure function (Xia et al., 2018). Changes in vasculature and inflammatory response in PE pathophysiology (Erez et al., 2022) might have been associated with COTL1 gene functional modulation, potentially linking it closely to vascular structure maintenance and inflammatory response regulation in PE. RBM39 was involved in transcription co-regulation and alternative RNA splicing, having core functions in various biological processes like vascular function, immune response, and cell signaling. Existing research had revealed possible associations between PE and abnormal HLA-G expression and RNA splicing (Djurisic et al., 2015), hence RBM39 might have been related to the complex pathophysiology of PE. The NINJ1 gene encoded a neural protein essential in controlling cell proliferation, migration, and invasion, especially in trophoblastic cells, where its knockdown might have promoted these processes but inhibited apoptosis (Zhang et al., 2022). Trophoblastic cells played a key role in placental development, and controlling their proliferation and migration was vital for pregnancy health (Marsh et al., 2022). PE was associated with placental dysfunction, and abnormal proliferation, migration, and apoptosis of the placenta were intimately related to disease processes. Therefore, NINJ1 might have had a direct association with the onset and development of PE, and its knockdown in trophoblastic cells might have triggered placental abnormalities, disrupted normal oxygen and nutrient supply, and induced clinical manifestations of PE. It was important to emphasize that these gene associations with PE risk do not necessarily imply causality. It was important to emphasize that these gene associations with PE risk do not necessarily imply causality. However, by analyzing these genes’ functions in cellular processes, we could unveil potential biological pathways, laying the groundwork for a deeper understanding of the pathogenic mechanisms of PE as well as potential intervention strategies.

eQTL and pQTL analyses were two distinct methods targeting gene expression and protein levels, respectively. Although both could be utilized to detect associations between specific gene variants and phenotypes, inconsistencies might have arisen due to their involvement in different biological processes and analytical scopes (Wei et al., 2023). While extracting corresponding cis-pQTL data from previously identified eQTL-positive genes, we found no evidence of causality. This inconsistency might have stemmed from eQTL analysis focusing on changes at the mRNA level, while pQTL analysis was influenced by post-transcriptional modifications, degradation, and other processes. Some post-transcriptional modification processes might have altered the efficiency with which mRNA was translated into protein, resulting in unobservable effects at the protein level (Li et al., 2023). Additionally, sample heterogeneity might also have led to inconsistencies between eQTL and pQTL analyses, as different samples exhibited biological and environmental variations (Wang et al., 2020). Therefore, such inconsistencies might reflect the complexity and multi-layered regulation of biological systems.

In our study, retinoic acid had attracted significant attention due to its potential interactions with several key genes. Combining existing literature, the relationship between retinoic acid and PE was particularly noteworthy. Prior research had emphasized the role of cell differentiation and growth in the mechanisms of PE (Xu et al., 2020), suggesting that genes regulated by retinoic acid might have been involved in PE development. Retinoic acid’s modulation of these genes could have offered novel avenues for the treatment and prevention of PE. For instance, IFITM3, a member of the IFITM family, participated in adaptive immunity (Yánez et al., 2020), while CD69 regulated immunity through Th/Treg cell balance (Gorabi et al., 2020). Recent studies had highlighted the association between aberrant immune responses, including adaptive and innate immunity, and PE (Lu and Hu, 2019). IFITM3 might have influenced PE through adaptive immune modulation, and CD69’s Treg balance mechanism could have been implicated in PE pathophysiology. By modulating these genes, retinoic acid could have engaged in immune regulation, potentially mitigating the risk of PE—an enticing hypothesis. However, while our study had offered promising preliminary insights, elucidating the precise mechanisms and potential therapeutic applications of retinoic acid in PE necessitated further *in vitro* and *in vivo* investigations.

The experiment had presented multiple advantages, primarily the ability of MR to mitigate many confounders inherent in observational studies due to the random genetic allocation at conception (Bowden and Holmes, 2019). Additionally, combining MR with single-cell data had offered insights into potential causal genetic-phenotypic relationships at the cellular level (Ziegenhain et al., 2017). The amalgamation of eQTL, pQTL, GWAS, and scRNA-seq data had further enhanced multi-faceted analyses. Nevertheless, certain limitations were evident. The analysis had predominantly targeted blood tissue expressions, potentially overlooking variations in other PE patient tissues. Its sole reliance on European population genetic datasets might have narrowed its inferential scope, and the exclusive use of summary statistics could have limited deeper exploration of specific causal relationships, such as differentiating early from late-onset PE or investigating non-linear associations. Furthermore, potential sources of bias or confounding in the MR analysis and the generalizability of the findings to other populations or ethnic groups should be discussed in more detail to provide a more comprehensive understanding of the study’s limitations.

## 5 Conclusion

Utilizing both MR and scRNA-seq techniques, this study highlighted significant cellular differences, especially within Th cells, and pinpointed IFITM3, NINJ1, COTL1, CD69, and YWHAZ as key genes implicated in PE’s pathogenesis. Despite certain limitations, these identified genes offered insight into PE’s fundamental mechanisms. Retinoic acid emerged as a potential therapeutic intervention, presenting promising avenues for treatment. Future research should validate these findings across diverse populations and explore the therapeutic potential of identified targets.

## Data Availability

The original contributions presented in the study are included in the article/Supplementary Material, further inquiries can be directed to the corresponding authors.
